# Work Climate, Improved Communication, and Cohesive Work Linked with Patient Safety Culture: Findings from a Sports Medicine Hospital

**DOI:** 10.3390/healthcare11243109

**Published:** 2023-12-06

**Authors:** Syed Sajid Ahmed, Samantha Poblete van Rijswijk, Abdulaziz Farooq

**Affiliations:** 1 Quality Management, Aspetar, Orthopaedic and Sports Medicine Hospital, Doha 29222, Qatar; 2 Rehabilitation Department, Aspetar, Orthopaedic and Sports Medicine Hospital, Doha 29222, Qatar; samantha.poblete@aspetar.com; 3 Research and Scientific Support, Aspetar, FIFA Medical Centre of Excellence, Orthopaedic and Sports Medicine Hospital, Doha 29222, Qatar; mohammed.farooq@aspetar.com

**Keywords:** patient safety, organizational culture, quality improvement, accreditation, sports medicine hospital

## Abstract

Background: This study aims to investigate the patient safety culture at a sports medicine hospital and explore the quality of healthcare and associated factors. Methods: In a cross-sectional study design, the Hospital Survey on Patient Safety Culture (HSOPC) tool was administered online among staff at a sports medicine hospital in Doha, Qatar. Out of 898 staff who received an email invitation, 504 participated (56.1%). Results: The results showed that 48.0% of the staff rated the patient safety grade as excellent and 37.5% as very good, totaling 85.5%. Factors associated with excellent or very good patient safety grades were management support OR 4.7 95% CI (1.8 to 12.3); team communication OR 3.0 95% CI (1.4 to 6.3), supervisor action supporting patient safety OR 3.5 95% CI (1.7 to 7.0) and other items related to work area such as working together: OR 3.0 95% CI (1.2 to 7.6), helping out busy areas OR 2.5 95% CI (1.1 to 5.5) and having good procedures and systems: OR 2.8 95% CI (1.4 to 5.8). Conclusions: Addressing management support, enhancing communication, and cohesive work within the work area facilitates a culture of trust that improves patient safety grades.

## 1. Introduction

Patient safety has been a key topic on the quality improvement agenda of healthcare organizations since the beginning of the new millennium. Public and professional concerns over patient safety, medical errors and adverse events have continued to increase. Therefore, these areas have rightly been used as fundamental criteria for healthcare accreditation processes [1]. The culture of patient safety is one of the key factors that determine safety and quality in healthcare organizations [2]. A culture of safety is built by awareness, knowledge, and a high level of perception of patient safety that all staff members share in a healthcare organization [3]. A culture of patient safety is an important healthcare quality dimension and is of high concern globally [4,5]. A culture of patient safety is supported by management and staff values, beliefs, norms, behaviors and attitudes. It also depends on the processes and procedures that reward and educate staff in relation to patient safety. 

A Canadian Adverse Event study [6] estimated that 7.5% of all admissions involve an adverse event in Canadian hospitals. This translates to 185,000 incidents, with an estimated 70,000 being potentially preventable. A 2016 systematic review commissioned by the World Health Organization identified missed and delayed diagnoses and medication errors as being the chief safety priorities in ambulatory care [7]. Missed and delayed diagnoses are relevant to sports medicine hospitals, where the primary concern is to assist athletes in returning to play or training as soon as possible.

Establishing a culture of patient safety in healthcare is essential to improve the quality of care and patient outcomes [8]. The creation of an organizational culture that encourages reporting, avoids blame and enhances communication is important to improve patient safety [9]. To make improvements in patient safety, it is important for healthcare organizations to assess the status of their existing culture in relation to patient safety and determine areas of priority to target for improvement [10]. Deploying a survey tool, such as the Agency for Healthcare Research and Quality Hospital Survey on Patient Safety Culture (HSOPSC), can support quality improvement by increasing staff awareness of safety-related issues. It is a rigorously designed tool designed to measure the culture of patient safety [11]. Previous studies in this domain have suggested that a culture of patient safety improves the quality of care, prevents errors, improves patient outcomes and reduces healthcare costs [12]. As the demand for quality in healthcare grows, healthcare organizations are faced with an increased need to establish a culture of patient safety [13]. 

Having an awareness of the factors that contribute to patient safety can help organizations to establish and improve their culture. Understanding the overall patient safety grade based on patient safety culture surveys helps organizations benchmark their performance internally and with external healthcare organizations. Emphasis is placed on achieving an “Excellent” or “Very Good” grade when assessing the overall climate of patient safety in these organizations. Regular patient safety culture tool deployment helps compare organizational performance to previous data. Identifying strengths helps sustain performance in these aspects and areas for improvement and facilitates action planning to improve performance in these specific areas. 

There are no known studies that have evaluated factors associated with a culture of patient safety in a sports medicine hospital. It is to our knowledge this is the first study that has been published in Qatar on the culture of patient safety, and this study will contribute to the growing evidence on the importance of developing a culture of patient safety. The structure of a sports medicine hospital is usually different from a general hospital. Patients are mostly athletes, and there is a substantial component of ambulatory care and rehabilitation. Sports surgery facilities are available, and the scope of practice is restricted to sports-related injuries and treatment. The priority of the organization is to assist athletes to return to play or training at full potential as soon as possible.

This study aims to report on the patient safety culture and compare the patient safety grade with other settings. The overall objective of this study is to determine the factors that influence the grading of patient safety at a sports medicine hospital.

## 2. Materials and Methods

A cross-sectional study design was employed to achieve the study objectives and assess the safety culture in the Aspetar institution. 

Aspetar is a specialized orthopedic and sports medicine hospital that has a key focus on the medical treatment of sports-related injuries. The structure of the hospital is composed of key medical departments, including sports medicine, musculoskeletal medicine, sports surgery, rehabilitation, sports dentistry, radiology, laboratory, sports podiatry, sports psychology, sports nutrition, pharmacy, research and medical clinics. This is linked with sports clubs and federations in Qatar. The hospital is staffed by more than 750 employees, around 80% of whom are healthcare professionals. There is an inpatient facility with 22 beds. Daily patient visits are about 400 per day, and the average number of surgeries per year is around 2000. 

The survey tool used in this study is version 1 of HSOPSC [14], developed by the Agency for Health Research and Quality to assess safety culture. We have used the original English and translated Arabic version of the HSOPSC tool, which was found to be reliable and valid in hospital settings [15].

The HSOPSC consists of 42 items categorized into 12 dimensions. These dimensions or composites are the following: teamwork within units, supervisor/manager expectations and actions promoting patient safety, organizational learning and continuous improvement, management support for patient safety, feedback and communication about errors, overall perceptions of patient safety, frequency of events reported, communication openness, teamwork across units, staffing, handoffs and transitions, and nonpunitive response to errors. Seven dimensions measure safety culture at the unit or departmental level. Three dimensions measure safety culture at the hospital level. The questionnaire also includes four outcome variables: the frequency of events reported, overall perception of safety, patient safety grade and number of events reported. Most questions ask staff to give agreement or frequency answers, using a Likert scale from 1 (“strongly disagree” or “never”) to 5 (“strongly agree” or “always”). Questions are both positively and negatively worded to reduce response bias. There are also two additional items related to the participant’s overall grade on patient safety for their work area/unit and to indicate the number of near misses and adverse events they have reported over the past 12 months.

The HSOPSC survey tool is administered online bi-annually to all clinical and nonclinical staff at this hospital. The survey is bilingual and delivered in English and Arabic. The questionnaire took around 15 min to complete. Additional data were collected that related to participants’ work area (clinical/nonclinical), background, years of experience in their profession, experience in the work unit, experience in the same hospital and workload (hours worked per week). In 2018, 319 responses were recorded, and an additional 185 responses were collected in 2020 and 2022 from staff who did not participate in the previous surveys. This was done to ensure unique participants were only included in the study. The factors assessed in relation to patient safety include the safety culture at the hospital unit where the staff worked, supervision, communications, frequency of events reported, patient safety grade awarded, hospital safety culture and number of events reported. 

The tool is an Accreditation Canada International requirement for the deployment of the Qmentum International accreditation program [1]. The demographic sections were identical to the original survey and were administered online. Emails were sent to staff to access the survey through a web link. The surveys were deployed over a three-week period. The survey was advertised by email, and staff were asked to participate. Contracted third-party staff were excluded from the study. Two email reminders were sent to the participants in the period corresponding to one week before the closing date. Participation was anonymous, and no personal information was collected that could identify the participants by name. Staff were provided with access to laptops to complete the tool online during their work hours. We provided contact information for participants to seek clarification on any questions. This study received ethical approval from Anti-Doping Lab Qatar (E2017000210). The confidentiality and anonymity of responses were maintained, and participants consented to participate in the online survey. 

All statistical analysis was performed using SPSS (Statistical Package for Social Sciences) v 21.0. The categorical variables were presented as counts and percentages. Dimension scores for each collaborative factor group were generated based on a four-step process; the “Strongly agree” and “Agree” responses were identified for each question and indicated a positive response. For the questions that were reversed, a positive response was indicated with an answer of “Disagree” or “Strongly disagree”. The percentage of positive results was calculated for each collaborative factor, and dimension scores were calculated as the average percentage of positive and negative responses for each question within each of the sections (Work Area/Supervisor/Communication/Frequency of Reporting and Hospital). 

A chi-square test was performed to compare items of the tool to the binary categorical variable overall patient safety grade (very good/excellent vs. failing/poor/acceptable). Items that were significant were considered for logistic regression analysis with overall patient safety and outcome variables and significant factors as potential covariates. Odds ratios with a 95% CI were reported, and *p*-value < 0.05 was considered as the cut-off for statistical significance.

## 3. Results

From the total of 898 staff, 504 participated and provided completed forms. They gave a response rate of 56.1%. The response rate was higher among clinical staff at 373 out of 504 (73.2%) compared to nonclinical staff, where 131 out of 394 (33.0%) completed the survey. Overall, the positive staff rating for the hospital patient safety grade stood at 85.5%, which included 48.0% as excellent and 37.5% as very good. Only 13.1% rated patient safety grade as acceptable, 0.8% as poor and 0.6% as failing. Of the 131 non-clinical staff that completed the survey, 87.8% reported a better patient safety grade (very good/excellent) when compared to clinical staff (316/373, 84.7%). 

### 3.1. Staff Characteristics

The length of employment in their current role did not impact the overall patient safety grading significantly. Staff who had worked in the hospital for ≤5 years (83.9%) provided similar ratings on the patient safety grade than staff who had worked in the hospital for more than 5 years (86.3%) (*p* = 0.509) (Table 1). Hours of work per week at the hospital did not influence the patient safety grading (*p* = 0.547).

There was no statistical difference between staff that did not provide direct patient care (88.2%) rating the patient safety grade as very good/excellent compared with staff who provided direct care to patients (84.4%) (*p* = 0.383). Employees who had less than five years of experience in their profession (90.3%) rated the patient safety grade as very good/excellent, which was similar when compared with staff that had more than five years of experience (84.7%) (*p* = 0.336). Staff that reported events in the past were no different compared to staff that did not report events on overall patient safety grade ratings (*p* = 0.300).

### 3.2. Work Area/Unit

**Figure 1:**
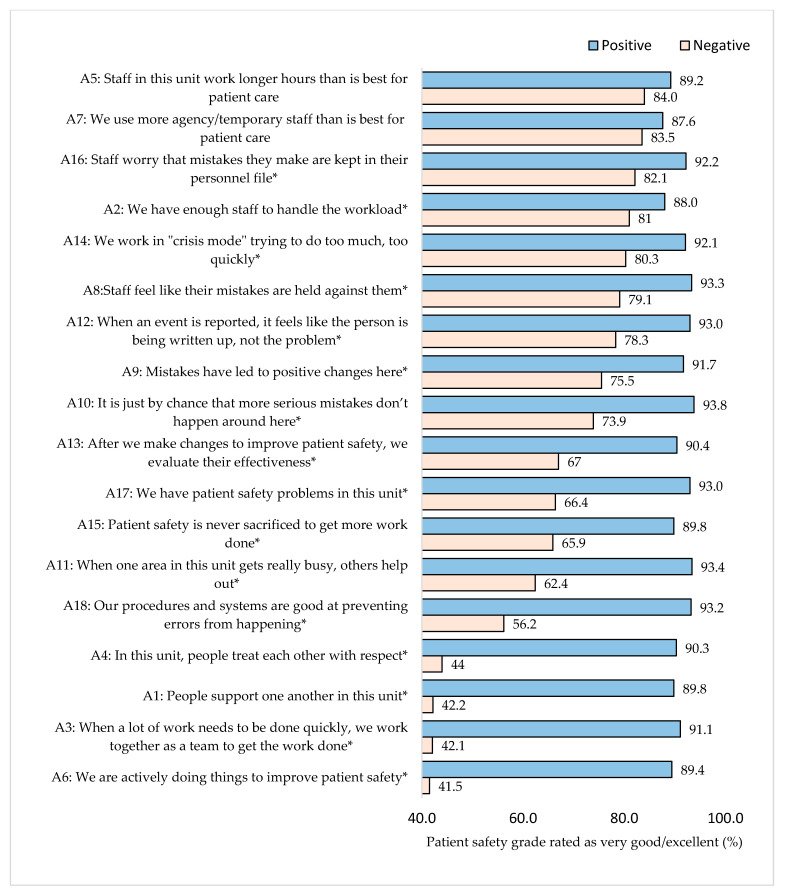
Percentage of overall patient safety grade (very good/excellent) based on positive or negative feedback related to the work area/unit. * Significant difference between positive and negative groups *p* < 0.05.

Participants who were positive that people support one another in their unit gave a positive rating for safety culture (89.8%), which was higher compared to those who were negative about people supporting one another in their unit (42.2%) (*p* < 0.001) (Figure 1). Participants who supported working together as a unit to complete work quickly, treating each other with respect and actively doing things to improve patient safety provided a better patient safety grade at the hospital (*p* < 0.001) compared to those participants who did not consider these items as important. Participants who did not report patient safety problems on their unit provided a better patient safety grade for the hospital (93.0%) compared to those who believed that there were problems in their unit (66.4%) (*p* < 0.001). Staff who believed their mistakes were held against them were more likely to report higher overall patient safety grade ratings (93.3%) compared to those who did not (79.1%) (*p* < 0.001). There were no statistically significant differences among staff who perceived that their unit works longer hours and uses more agency/temporary staff than is best for patient care compared to staff who did not hold this perception. 

### 3.3. Supervisor

**Figure 2:**
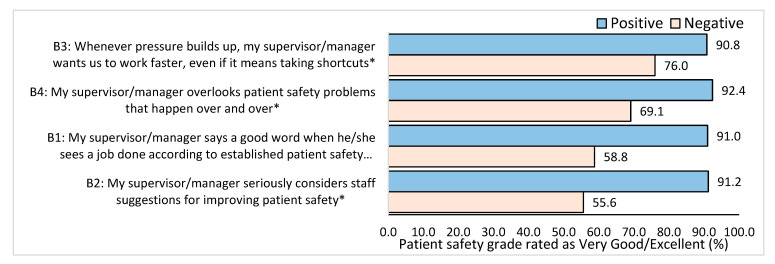
Percentage of overall patient safety grade (very good/excellent) based on positive or negative feedback related to feedback on supervisor/manager. * Significant difference between positive and negative groups *p* < 0.05.

Staff who reported that their supervisor appreciated their work related to patient safety practices awarded the hospital a better patient safety rating (91.0%) compared to those participants who reported that supervisors did not appreciate their work (58.8%) (*p* < 0.001) (Figure 2). Staff whose supervisor seriously considered staff suggestions for improving patient safety awarded the hospital a better patient safety grade (91.2%) than the supervisor who did not (55.6%) (*p* < 0.001). Participants who reported that their supervisor did not support any shortcuts at work that impact patient safety were awarded a better patient safety grade (90.8%) than those who had supervisors who supported shortcuts (76.0%) (*p* < 0.001). Similarly, staff with a supervisor who did not overlook patient safety problems were awarded a better patient safety grade (92.4%) than staff whose supervisor did overlook patient safety problems (69.1%) (*p* < 0.001).

### 3.4. Communication 

**Figure 3:**
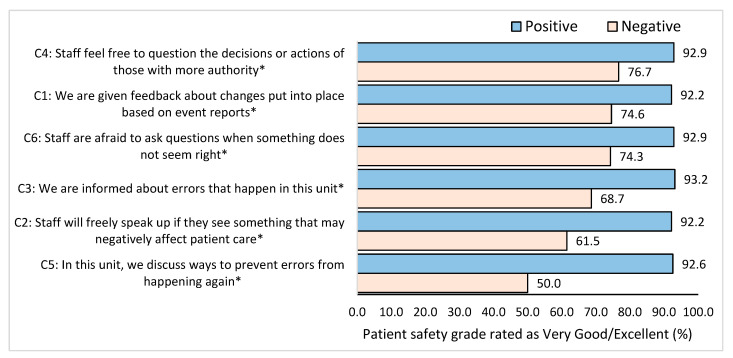
Percentage of overall patient safety grade (very good/excellent) based on positive or negative feedback related to feedback on communication. * Significant difference between positive and negative groups *p* < 0.05.

Out of the six items for HSOPC related to communication, the item concerning staff discussions on ways to prevent errors from happening again in their units was significant (*p* < 0.001). The staff who reported positively on this item awarded a higher patient safety grade (92.6%) compared to staff who did not (50.0%) (*p* < 0.001) (Figure 3). Furthermore, when staff are given feedback, informed about errors, and allowed to speak freely and ask questions, they are more likely to give a higher overall patient safety grade rating (*p* < 0.001). 

### 3.5. Frequency of Events Reported

**Figure 4:**
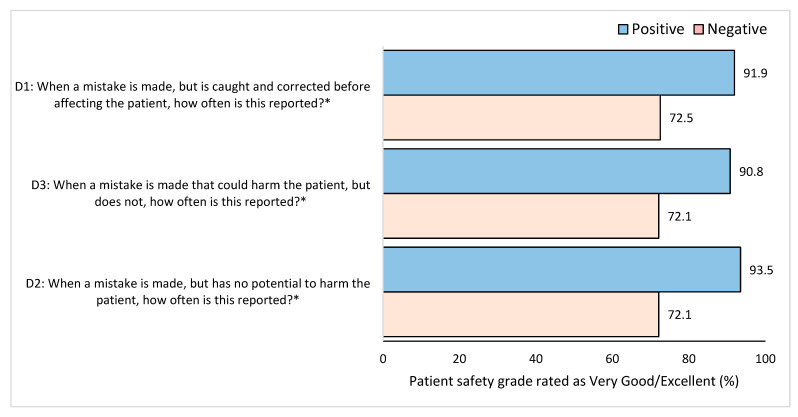
Percentage of overall patient safety grade (very good/excellent) based on positive or negative feedback related to the frequency of events reported. * Significant difference between positive and negative groups *p* < 0.05.

Staff that reported a mistake most of the time or always, even when there was no potential for patient harm, were awarded a better patient safety grade than staff that did not in all three items (*p* ≤ 0.001 (Figure 4)). 

### 3.6. Hospital

**Figure 5:**
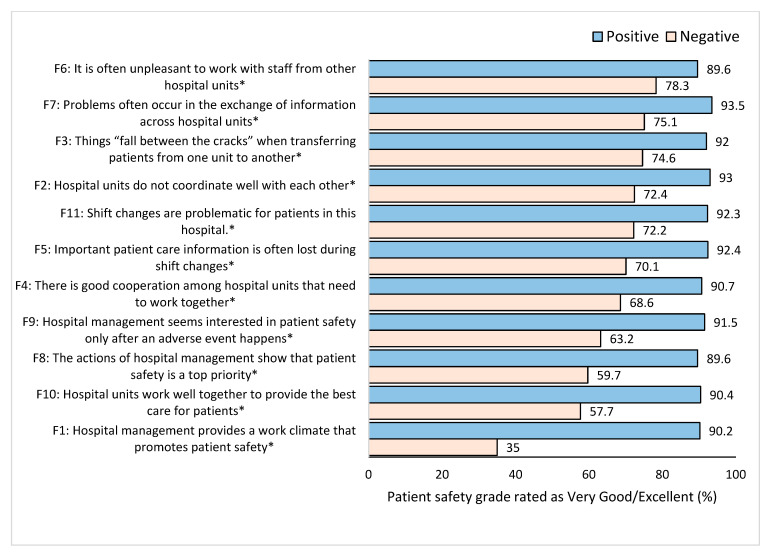
Percentage of overall patient safety grade (very good/excellent) based on positive or negative feedback related to the hospital. * Significant difference between positive and negative groups *p* < 0.05.

Staff that reported that the hospital management provides a work climate that promotes patient safety provided a better patient safety grade (90.2%) than staff that did not (35.0%), *p* < 0.001 (Figure 5). When participants perceived that hospital units coordinated well with each other, they provided a higher patient safety grade (93.0%) compared to those who perceived that they did not coordinate well with each other (72.4%) (*p* < 0.001). Participants who reported that patient care information was not lost during shift change also provided a higher rating on the overall patient safety grade (92.4%) compared to those who reported that patient care information was lost during shift changes (70.1%). (*p* < 0.001). Staff who believed that actions by hospital management showed that patient safety was a top priority rated higher on the overall patient safety grade (89.6% compared to 59.7%, *p* < 0.001). Staff who disagreed that hospital management was only interested in patient safety after an adverse event were more likely to provide a higher overall patient safety grade (91.5% vs. 63.2%, *p* < 0.001). 

The logistic regression analysis revealed that the perception that hospital management provided a work climate that promotes patient safety was associated with increased odds for a very good/excellent overall patient safety grade (OR = 4.7, *p* = 0.002) (Table 2). Staff who reported that when a lot of work needed to be done quickly, they came together as a team to achieve this task were more likely to award a very good/excellent patient safety grade (OR = 3.0, *p* = 022). Three other items related to work areas, such as helping each other when a unit gets busy, perceiving an absence of patient safety problems, and procedures/systems rated good at preventing errors, were strongly associated with positive patient safety grade (OR = 2.5, *p* = 0.021; OR = 2.1, *p* = 0.035 and OR = 2.8, *p* = 0.005 respectively). Staff who reported that supervisors did not overlook recurring patient safety problems were more likely to give a positive patient safety grade (OR = 3.5, *p* < 0.001). Respondents who reported that staff always discussed ways to prevent errors from happening again were more likely to report a better patient safety grade. (OR 3.0, *p* = 0.004). When staff agreed that there were no patient safety problems in their unit, this was associated with them reporting a higher patient safety grade (OR = 2.1, *p* = 0.035). 

## 4. Discussion

### 4.1. Statement of Principal Findings

**Figure 6:**
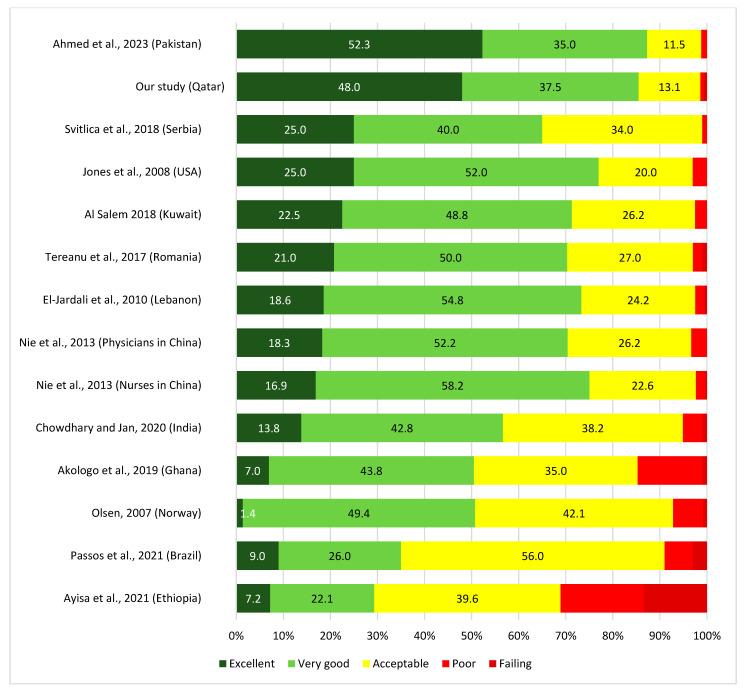
Overall patient safety grade in our study compared to other studies in hospitals [15,16,18,19,20,21,22,24,25,26,28,29].

This study reported that at a sports medicine hospital, the overall patient safety grade, as assessed by staff, was 85.5% (Excellent or Very Good). The percentage of ‘excellent or very good’ overall patient safety grade achieved in this study is better than most of the similar studies that assessed the patient safety grade at 21 critical access hospitals in the USA (77.0%) [16], 60.0% at 13 general hospitals in Saudi Arabia [17], 70.3% at 3 public hospitals in Kuwait [18], 70% at 68 hospitals in Lebanon [15], and 73.0% at 32 hospitals in 15 cities in China [19], (71% among hospital staff in six hospital across four regions in Romania [20] 87.3% based on a recent study of staff at University Hospital in Pakistan [21], 29.3% among healthcare providers in a specialised hospital in Northwest Ethiopia [22], 20.0% among nurses in ICUs from Egypt [23], 35.0% among health care professionals from a public hospital in Brazil [24], 50.8% among healthcare providers in the Upper East region of Ghana [25], 65% among healthcare workers in Serbian setting [26], 58% among Finland forensic psychiatric hospital staff [27], 50.8% in Norwegian university hospital [28], 56.6% in Tertiary care hospital in North India [29] (Figure 6). The variation in the prevalence of patient safety grade could be attributed to the size of the hospital settings. From the findings above and confirmed from a study in Kuwait [30], it appears that staff from larger hospital settings provided lower grades on patient safety compared to smaller hospitals. 

### 4.2. Interpretation within the Context of the Wider Literature

Upon further analysis of our data, there was a statistically significant association between the selected factors that were associated with ‘Excellent/Very Good’ patient safety grade. In our study, we noticed that when the hospital management provides a work culture that promotes patient safety, there is a significant improvement in patient safety grade provided to the hospital, and this is evidence of a just culture [16]. 

When a lot of work needs to be done quickly, “we work together as a team to get the work done” shows the willingness of staff to work on common goals, which is evidence of a flexible culture. Teamwork within work areas/units is critical to ensure that there is improved safety in their work area/unit [31]. Higher scores on teamwork increase the likelihood of participants reporting a higher patient safety grade consistent with other studies [32,33]. Staff who reported that their mistakes are held against them were more likely to report higher overall patient safety grade ratings, suggesting that an environment where healthcare providers are accountable for quality of care will lead to improved patient safety [34]. 

Good communication with and across healthcare teams is the key to mitigating any threats to patient safety [15]. Results from our study show a significant association between open discussions on patient safety and a positive patient safety grade [35]. When there is discussion in teams on ways to prevent errors from happening again, this shows a positive patient safety culture and is evidence of a learning culture. 

When staff confirm that they do not have safety problems on their teams, they are awarded a positive patient safety grade. This is evidence of an informed culture agreeing with similar studies in Lebanon [15,33] and Oman [32].

When staff say that important patient care information is rarely lost during shift changes, it is evidence of an informed culture [16]. Higher scores on handoffs and transitions increased the likelihood of having a better perception of safety among participants and the likelihood of these participants being awarded a better patient safety grade. 

The results regarding factors influencing the patient safety grade are consistent with previous published research, which demonstrated that the safety culture varies by position and work area [31,32]. Specifically, this relates to staff that do not deliver direct patient care, rating the patient safety grade higher than staff that deliver direct patient care [16]. This may be due to a perception of a punitive culture [11,36,37,38,39].

In our study, there were high odds of patient safety grade (OR = 3.5, *p* < 0.001) when supervisors did not overlook frequently occurring problems in their units. Similar relationships were reported among registered nurses working in government hospitals across Oman [40], as well as in a national survey of healthcare practitioners across hospitals in Sweden [41] and hospital staff from Romania [20]. Both of these studies emphasized supervisors’/managers’ expectations and actions promoting patient safety were major predictors of patient safety culture. In agreement with our findings, a most recent study that assessed the Local Leadership Score (LLS) among staff across 31 Midwestern hospitals in the US showed that the LLS score was positively associated with several domains including safety climate and teamwork climate [42]. A previous study among nurses in Canada that investigated the impact of leadership, interactional justice, and work environment on patient safety supported that positive leader–staff relationships lead to safer work climates [43]. 

Overall, the results from this study show strong evidence of a growing interest among healthcare organizations to assess the safety culture and use it as a tool for improvement [44].

### 4.3. Implications for Policy, Practice and Research

The study contributes to the growing evidence that the establishment of a culture of patient safety is important to move the organization across the quality continuum. There are important implications for practice, including a positive attitude towards patient safety by staff improves the patient safety grading of a hospital. It is vital to improve teamwork between units to improve patient safety. Further analysis is recommended to identify the presence of microcultures within organizations so that customized interventions can be implemented. 

### 4.4. Strengths and Limitations

This is the first study of patient safety grades in a Sports Medicine Hospital setting. Although the data collected from participants had very few and incomplete responses, there were a few limitations to consider. The response rate was low (36%); however, it is generally accepted that web-based has a lower response than in-person surveys [45]. The web-based surveys also provide more anonymity compared to face-to-face interviews, where there is a risk of identification and external influence.

## 5. Conclusions

The overall patient safety grade achieved in this study is significantly better than similar studies that assessed the patient safety grade in hospitals. To create a culture of safety and improvement, healthcare leaders must create a climate of open communication for staff within their own work areas, as well as in the overall organization, as these are key factors that influence the overall patient safety grade [46]. Ensuring patients’ quick return to play involves a team with members from multiple disciplines. Hence, a high performance and safety culture can enhance facilitating teamwork [44]. Consistent with our findings, emphasis must be placed on reducing punitive responses to error and having supportive supervisors to improve safety culture [11,36,38,39].

Essentially, the deployment of the HSOPSC on a regular basis helps to measure the patient safety pulse of an organization and identify and make relevant improvements [47]. The results of this study can be used to plan targeted interventions. Leaders can use the data and facilitate a culture of trust that encourages two-way communications across healthcare organizations. 

## Figures and Tables

**Table 1:** Overall patient safety ^1^ grade based on background information.

Variables	Failing/Poor/Acceptable n (%)	Very Good/Excellent n (%)	*p*-Value
	n = 73	n = 431	
How long have you worked in this hospital?			
≤5 years	24 (12.6)	167 (87.4)	0.363
>5 years	49 (15.7)	263 (84.3)	
How long have you worked in your current hospital work area/unit?			
≤5 years	29 (16.1)	151 (83.9)	0.509
>5 years	44 (13.7)	278 (86.3)	
Typically, how many hours per week do you work in this hospital?			
<20 h	0 (0.0)	7 (100.0)	
20–39 h	23 (14.7)	133 (85.3)	0.547
40–59 h	50 (14.7)	290 (85.3)	
Staff			
Clinical	57 (15.3)	316 (84.7)	0.471
Non-Clinical	16 (12.2)	115 (87.8)	
In your staff position, do you typically have direct interaction or contact with patients?			
Yes	58 (15.6)	314 (84.4)	0.383
No	15 (11.8)	112 (88.2)	
How long have you worked in your current specialty or profession?			
≤5 years	6 (9.7)	56 (90.3)	0.336
>5 years	67 (15.3)	371 (84.7)	
Reporting events during the last 12 months			
Yes	21 (17.5)	99 (82.5)	0.300
No	52 (13.6)	331 (86.4)	
Reporting events during the last 12 months			
None	52 (13.6)	331 (86.4)	
1–2	15 (23.8)	48 (76.2)	
3–5	3 (9.7)	28 (90.3)	0.281
6–10	3 (15.0)	17 (85.0)	
11–20	0 (0.0)	2 (100.0)	
>=21	0 (0.0)	4 (100.0)	

^1^ Patient safety grade is grouped as (failing/poor/acceptable or very good/excellent) based on rating.

**Table 2:** Overall patient safety grade based on background information.

Variables	Odds Ratio	95% CI	*p*-Value
A3. When a lot of work needs to be done quickly, we work together as a team to get the work done	3.0	1.2–7.6	0.022
A11. When one area in this unit gets really busy, others help out	2.5	1.1–5.5	0.021
A17. We do not have patient safety problems in this unit	2.1	1.1–4.3	0.035
A18. Our procedures and systems are good at preventing errors from happening	2.8	1.4–5.8	0.005
B4. My supervisor/manager does not overlook patient safety problems that happen over and over	3.5	1.7–7.0	0.000
C5. In this unit, we discuss ways to prevent errors from happening again	3.0	1.4–6.3	0.004
F1. Hospital management provides a work climate that promotes patient safety	4.7	1.8–12.3	0.002

## Data Availability

The datasets used and/or analyzed during the current study are available from the corresponding author on request.

## References

[1] Accreditation Canada Qmentum Accreditation Program. https://accreditation.ca/accreditation/qmentum/.

[2] Alex Kim R.J., Chin Z.H., Sharlyn P., Priscilla B., Josephine S. (2019). Hospital survey on patient safety culture in Sarawak General Hospital: A cross sectional study. Med. J. Malays..

[3] Huong Tran L., Thanh Pham Q., Nguyen D.H., Tran T.N.H., Bui T.T.H. (2021). Assessment of Patient Safety Culture in Public General Hospital in Capital City of Vietnam. Health Serv. Insights.

[4] Wami S.D., Demssie A.F., Wassie M.M., Ahmed A.N. (2016). Patient safety culture and associated factors: A quantitative and qualitative study of healthcare workers’ view in Jimma zone Hospitals, Southwest Ethiopia. BMC Health Serv. Res..

[5] Pronovost P.J., Berenholtz S.M., Goeschel C.A., Needham D.M., Sexton J.B., Thompson D.A., Lubomski L.H., Marsteller J.A., Makary M.A., Hunt E. (2006). Creating High Reliability in Health Care Organizations. Health Serv. Res..

[6] Baker G.R., Norton P.G., Flintoft V., Blais R., Brown A., Cox J., Etchells E., Ghali W.A., Hébert P., Majumdar S.R. (2004). The Canadian Adverse Events Study: The incidence of adverse events among hospital patients in Canada. Cmaj.

[7] Panesar S.S., deSilva D., Carson-Stevens A., Cresswell K.M., Salvilla S.A., Slight S.P., Javad S., Netuveli G., Larizgoitia I., Donaldson L.J. (2016). How safe is primary care? A systematic review. BMJ Qual. Saf..

[8] Chen I.C., Li H.H. (2010). Measuring patient safety culture in Taiwan using the Hospital Survey on Patient Safety Culture (HSOPSC). BMC Health Serv. Res..

[9] Nygren M., Roback K., Öhrn A., Rutberg H., Rahmqvist M., Nilsen P. (2013). Factors influencing patient safety in Sweden: Perceptions of patient safety officers in the county councils. BMC Health Serv. Res..

[10] Nieva V.F., Sorra J. (2003). Safety culture assessment: A tool for improving patient safety in healthcare organizations. Qual. Saf. Health Care.

[11] Stoyanova R., Dimova R., Tornyova B., Mavrov M., Elkova H. (2021). Perception of Patient Safety Culture among Hospital Staff. Slov. J. Public Health.

[12] Ginsburg L., Gilin Oore D. (2016). Patient safety climate strength: A concept that requires more attention. BMJ Qual. Saf..

[13] Aljaffary A., Al Yaqoub F., Al Madani R., Aldossary H., Alumran A. (2021). Patient Safety Culture in a Teaching Hospital in Eastern Province of Saudi Arabia: Assessment and Opportunities for Improvement. Risk Manag. Healthc. Policy.

[14] Sorra J., Gray L., Streagle S. (2018). AHRQ Hospital Survey on Patient Safety Culture: User’s Guide.

[15] El-Jardali F., Jaafar M., Dimassi H., Jamal D., Hamdan R. (2010). The current state of patient safety culture in Lebanese hospitals: A study at baseline. Int. J. Qual. Health Care.

[16] Jones K.J., Skinner A., Xu L., Sun J., Mueller K., Henriksen K., Battles J.B., Keyes M.A., Grady M.L. (2008). Advances in Patient Safety: The AHRQ Hospital Survey on Patient Safety Culture: A Tool to Plan and Evaluate Patient Safety Programs. Advances in Patient Safety: New Directions and Alternative Approaches (Vol. 2: Culture and Redesign).

[17] Alahmadi H.A. (2010). Assessment of patient safety culture in Saudi Arabian hospitals. Qual. Saf. Health Care.

[18] Al Salem G.F. (2018). An Assessment of Safety Climate in Kuwaiti Public Hospitals. Ph.D. Thesis.

[19] Nie Y., Mao X., Cui H., He S., Li J., Zhang M. (2013). Hospital survey on patient safety culture in China. BMC Health Serv. Res..

[20] Tereanu C., Ghelase M.S., Sampietro G., Furtunescu F.L., Dragoescu A., Molnar A., Moraru D., Stanescu C., Gavrila O.A., Patrascu A. (2017). Measuring Patient Safety Culture in Romania Using the Hospital Survey on Patient Safety Culture (HSOPSC). Curr. Health Sci. J..

[21] Fasih Ali A., Fozia A., Tahir M., Muhammad Sohail H., Zehra Feroze A., Asim B., Hasnain Z., Asad L. (2023). Measuring the patient safety culture at a tertiary care hospital in Pakistan using the Hospital Survey on Patient Safety Culture (HSOPSC). BMJ Open Qual..

[22] Ayisa A., Getahun Y., Yesuf N. (2021). Patient Safety Culture and Associated Factors Among Health-Care Providers in the University of Gondar Comprehensive Specialized Hospital, Northwest Ethiopia. Drug Healthc. Patient Saf..

[23] Salem M., Labib J., Mahmoud A., Shalaby S. (2019). Nurses’ Perceptions of Patient Safety Culture in Intensive Care Units: A Cross-Sectional Study. Open Access Maced. J. Med. Sci..

[24] de Brito Passos A.C., dos Santos D.B., de França Fonteles M.M. (2021). Patient Safety Culture: From Perception to Assessment. J. Young Pharm..

[25] Akologo A., Abuosi A.A., Anaba E.A. (2019). A cross-sectional survey on patient safety culture among healthcare providers in the Upper East region of Ghana. PLoS ONE.

[26] Brestovački-Svitlica B., Milutinović D., Božić A., Maletin S., Lalić I. (2018). The assessment of patient safety culture-the psychometric study of the serbian version of the Questionnaire hospital survey on patient safety culture. Med. Pregl..

[27] Kuosmanen A., Tiihonen J., Repo-Tiihonen E., Eronen M., Turunen H. (2013). Patient safety culture in two Finnish state-run forensic psychiatric hospitals. J. Forensic Nurs..

[28] Olsen E. (2007). Workers’ perceptions of safety culture at a hospital. Tidsskr. Nor. Laegeforening Tidsskr. Prakt. Med. Ny Raekke.

[29] Chowdhary E., Jan F.A. (2020). Appraisal of Patient Safety Culture in a Tertiary Care Hospital from North India. J. Med. Sci. Clin. Res..

[30] Badr H.E., AlFadalah T., El-Jardali F. (2020). Towards promoting patient safety practices: Baseline assessment of patient safety culture in three private hospitals. Int. J. Healthc. Manag..

[31] Richter J.P., McAlearney A.S., Pennell M.L. (2016). The influence of organizational factors on patient safety: Examining successful handoffs in health care. Health Care Manag. Rev..

[32] Fuseini A.-K.J., Teixeira da Costa E.I.M., Matos F.A.S.d., Merino-Godoy M.-d.-l.-A., Nave F. (2023). Patient-Safety Culture among Emergency and Critical Care Nurses in a Maternal and Child Department. Healthcare.

[33] El-Jardali F., Dimassi H., Jamal D., Jaafar M., Hemadeh N. (2011). Predictors and outcomes of patient safety culture in hospitals. BMC Health Serv. Res..

[34] Lee S.-H., Phan P.H., Dorman T., Weaver S.J., Pronovost P.J. (2016). Handoffs, safety culture, and practices: Evidence from the hospital survey on patient safety culture. BMC Health Serv. Res..

[35] Alsabri M., Boudi Z., Lauque D., Dias R.D., Whelan J.S., Östlundh L., Alinier G., Onyeji C., Michel P., Liu S.W. (2022). Impact of Teamwork and Communication Training Interventions on Safety Culture and Patient Safety in Emergency Departments: A Systematic Review. J. Patient Saf..

[36] Khater W.A., Akhu-Zaheya L.M., Al-Mahasneh S.I., Khater R. (2015). Nurses’ perceptions of patient safety culture in Jordanian hospitals. Int. Nurs. Rev..

[37] Okuyama J.H.H., Galvao T.F., Silva M.T. (2018). Healthcare Professional’s Perception of Patient Safety Measured by the Hospital Survey on Patient Safety Culture: A Systematic Review and Meta-Analysis. Sci. World J..

[38] Quillivan R.R., Burlison J.D., Browne E.K., Scott S.D., Hoffman J.M. (2016). Patient Safety Culture and the Second Victim Phenomenon: Connecting Culture to Staff Distress in Nurses. Jt. Comm. J. Qual. Patient Saf..

[39] Ramos R.R., Calidgid C.C. (2018). Patient safety culture among nurses at a tertiary government hospital in the Philippines. Appl. Nurs. Res..

[40] Ammouri A.A., Tailakh A.K., Muliira J.K., Geethakrishnan R., Al Kindi S.N. (2015). Patient safety culture among nurses. Int. Nurs. Rev..

[41] Danielsson M., Nilsen P., Rutberg H., Årestedt K. (2019). A National Study of Patient Safety Culture in Hospitals in Sweden. J. Patient Saf..

[42] Tawfik D.S., Adair K.C., Palassof S., Sexton J.B., Levoy E., Frankel A., Leonard M., Proulx J., Profit J. (2023). Leadership Behavior Associations with Domains of Safety Culture, Engagement, and Health Care Worker Well-Being. Jt. Comm. J. Qual. Patient Saf..

[43] Squires M., Tourangeau A., Spence Laschinger H.K., Doran D. (2010). The link between leadership and safety outcomes in hospitals. J. Nurs. Manag..

[44] Azyabi A., Karwowski W., Davahli M.R. (2021). Assessing Patient Safety Culture in Hospital Settings. Int. J. Environ. Res. Public Health.

[45] Kwak N., Radler B. (2002). A comparison between mail and web surveys: Response pattern, respondent profile, and data quality. J. Off. Stat..

[46] Alsabri M., Castillo F.B., Wiredu S., Soliman A., Dowlat T., Kusum V., Kupferman F.E. (2021). Assessment of Patient Safety Culture in a Pediatric Department. Cureus.

[47] Gampetro P.J., Segvich J.P., Jordan N., Velsor-Friedrich B., Burkhart L. (2021). Perceptions of Pediatric Hospital Safety Culture in the United States: An Analysis of the 2016 Hospital Survey on Patient Safety Culture. J. Patient Saf..

